# Oxidative damage biomarkers and antioxidant enzymes in saliva of patients with peri-implant diseases

**DOI:** 10.1186/s40729-024-00562-x

**Published:** 2024-10-14

**Authors:** Yerda Özkan Karasu, Oğuzhan Maden, Cenk Fatih Çanakçı

**Affiliations:** Department of Periodontology, Faculty of Dentistry, Ataturk University, Erzurum, Turkey

**Keywords:** 8-hydroxydeoxyguanosine, Malondialdehyde, Superoxide dismutase, Glutathione peroxidase, Peri-implantitis, Peri-mucositis

## Abstract

**Objectives:**

8-hydroxydeoxyguanosine (8-OHdG) and Malondialdehyde (MDA) are commonly used as markers to evaluate oxidative DNA and Lipid damage in disorders including chronic inflammatory diseases. Superoxide dismutase (SOD) and glutathione peroxidase (GPx) protect tissues against oxidative injury from free oxygen radicals generated by various metabolic processes. The aim of this study was to evaluate 8-OHdG and MDA levels, and SOD and GPx activities in whole saliva of patients with peri-implant diseases.

**Materials and methods:**

A cross-sectional study was conducted on a sum of 60 age gender balanced; peri-implantitis (*n* = 20), peri-mucositis (*n* = 20) and healthy (*n* = 20) individuals. Unstimulated whole saliva samples were collected and to determine the clinical condition of each subject; the plaque index (PI), gingival index (GI), peri-implant probing pocket depth (PIPD), peri-implant presence of bleeding on probing (BOP) (with/without suppuration) and radiographic signs of crestal bone loss (BL) were measured. The salivary 8-OHdG level was measured using the ELISA method. SOD, GPx activities and MDA levels were determined spectrophotometrically.

**Results:**

A total of 60 individuals had evaluations of 318 implants. In comparison to the peri-mucositis and peri-implantitis groups, the healthy group had significantly lower PI and GI scores (*p* < 0.001). The PIPD value differed amongst the groups, with the peri-implantitis group having the highest value (*p* < 0.001). Compared to the peri-mucositis and control groups, the peri-implantitis group had a significantly higher BL score (*p* < 0.001 and *p* < 0.001, respectively). The peri-implantitis group showed a significantly higher 8-OHdG level (*p* < 0.001; *p* < 0.001 respectively) than the peri-mucositis and control groups. Compared to the peri-mucositis and control groups, the peri-implantitis group had a significantly higher MDA level (*p* < 0.001 and *p* < 0.001, respectively). The peri-implantitis group had a significantly higher SOD level (*p* < 0.001 and *p* < 0.001, respectively) in comparison to the peri-mucositis and control groups. There was no significant difference in GPx levels between the peri-mucositis and control groups (*p* > 0.05), while the peri-implantitis group had significantly lower GPx levels than the peri-mucositis and control groups (*p* < 0.001 and *p* < 0.001, respectively).

**Conclusions:**

Elevated levels of oxidative stress in saliva may indicate the onset of pathological bone loss surrounding the implant and may be an indication of peri-implantitis.

**Clinical relevance:**

In peri-implant diseases, changes may occur in the levels of 8-OHdG, MDA, SOD and GPx in saliva, which may lead to a deterioration in the oxidant/antioxidant balance.

## Introduction

Peri-implant diseases are a degenerative disorder that affects the tissues around dental implants, and it is defined by inflammation of the soft tissues surrounding the implants and a gradual destruction of supporting bone [1]. Peri-implantitis results from an imbalance between the bacterial load and the host response following proper osseointegration of an implant [2]. There is incomplete understanding of the histopathologic and clinical factors that cause peri-implant mucositis to change into peri-implantitis [3].

The term “oxidative stress” refers to the state that arises when the body’s natural equilibrium between antioxidants and oxidants is upset, potentially causing harm to the organism [4]. Increasing oxidative stress has the potential to harm multiple cellular and extracellular components, resulting in the development of cancer, aging, and numerous other illnesses [4, 5]. These harmful consequences of elevated oxidative stress are known as oxidative damage, and they emerge upon exposure to a comparatively high concentration of reactive oxygen species (ROS) and/or a reduction in the antioxidant defense system (AO) against ROS. In response to various antimicrobial stimuli during phagocytosis of periodontitis-associated bacteria, ROS can be generated by PMNs [6]. Furthermore, it is recognized that ROS have been affected in the pathophysiology of periodontitis [7, 8]. According to certain theories, PMNs are stimulated by bacterial antigens and as a result, create and release large amounts of ROS, which causes increased oxidative damage to alveolar bone and gingival tissue [9]. ROS are involved in DNA damage, lipid peroxidation (LPO), the oxidation of enzymes like anti-proteases, the depolymerization of extracellular matrix components, and the activation of pro-inflammatory cytokines [7]. Polymorphonuclear leukocytes (PMN) and macrophages were more common in the lesions at the locations of peri-implantitis, which were also dominated by lymphocytes and plasma cells, just like in periodontitis [10, 11]. The peri-implant description may also be associated with the modulation of multiple cytokines and chemokines in favor of a pro-inflammatory host response profile, as well as oxidative stress mechanisms and excessive production of reactive oxygen species, both of which have a significant impact on the host response [12].

All tissue and fluids in the body contain molecules of antioxidants [8, 13]. Tissues are shielded from oxidative damage by antioxidant enzymes like SOD and GPX, which neutralize free oxygen radicals produced by different metabolic processes [4, 5]. The GPX family of antioxidant enzymes oxidizes reduced glutathione or s-nitrosoglutathione to lower H2O2 and/or lipid hydrogen peroxides [4, 5]. Extracellular GPX (eGPX), which has a significant antioxidant role at numerous extracellular surfaces and spaces, and classical cellular GPX are the two types of GPX that are known.

Nucleoside oxidation is one type of DNA damage that can be brought on by oxidative stress. DNA repair results in the excretion of 8-hydroxydeoxyguanosine (8-OHdG), an oxidized nucleoside, in body fluids. Numerous investigations have revealed that 8-OHdG can function as a biomarker of oxidative stress in biological fluids. In fact, 8-OHdG is frequently employed as a marker to assess oxidative damage in disorders, such as chronic inflammatory diseases [9, 14]. Uncontrollably high ROS generation can also result in lipid peroxidations (LPO). The process of LPO happens when ROS interacts with polyunsaturated fatty acids in lipoproteins or cell membranes. One of the last by products of LPO’s breakdown, malondialdehyde (MDA) is an indication of LPO and has a number of harmful impacts on biological systems [9]. We hypothesized that there may be a disruption in the oxidant and anti-oxidant balance in peri-implant diseases. This study assessed the levels of MDA, 8-OHdG, and activity of SOD and GPx in the saliva of individuals with peri-implantitis and peri-mucositis, and the collected data were contrasted with those of healthy participants.

## Materials and methods

### Study selection

Those patients who applied for treatment due to peri-implant problems or for routine check-ups to the Ataturk University, Faculty of Dentistry, were included in this study. Based on the effect size f = 0.980 from the Sánchez-Siles et al. [15] study, the one-way anova method was used to calculate the sample size using G*power 3.1 indicated that sixty participants (twenty in each group) would be needed for the study at 80% power and 95% confidence level. All participants had at least 26 and at most 28 natural teeth, and these teeth were healthy with respect to periodontal disease. They had 4–6 implants, and these implants were placed in the posterior mandible/maxilla region. Also, at least two implants were in a single quadrant. A total of 60 participants were divided into three groups; 1) Peri-implantitis group 2)Peri-mucositis group 3) Healthy controls. All individuals in this study signed an informed consent to a research protocol that had been reviewed and approved by the ethics committee of Ataturk University, with the approval number B.30.2.ATA.0.01.00/463.

Inclusion criteria: being between 25 and 60 years of age; having at least 26 and at most 28 natural teeth, having 4, 5, or 6 implants in the posterior maxilla and mandible, with at least two implants in a single quadrant; being periodontally healthy; brushing teeth at least twice a day and did not use mouthwash; being systemically healthy.

Exclusion criteria were as follows: having less than 4 or more than 6 implants; mobility of any implant; having periodontitis or gingivitis; having active caries lesions, having received periodontal treatment within 6 months; having apical abscess or gingival/periodontal abscess; having oral mucosal diseases; brushing teeth less than 2 times a day; using mouthwash; smoking at any point in life; ongoing orthodontic treatment; any systemic disease (diabetes, cardiovascular diseases, thyroid, etc.); having salivary gland disease; using antibiotics, NSAIDs, corticosteroids, or oral contraceptives within the last 3 months; pregnancy; and history of osteoporosis or radiotherapy to the cervicofacial region.

### Clinical Periodontal measurements

For all groups, using a periodontal probe (Williams, Hu-Friedy, Chicago, IL), six sites (mesio-buccal, buccal, disto-buccal, lingual, and disto-lingual) of every tooth—aside from third molars—were assessed for the Silness-Löe plaque index (PI), bleeding on probing (BOP), probing depth (PD), and clinical attachment level (CAL). Periodontal examination was performed by one examiner (YÖK).

*Peri-implantitis group*: Peri-implantitis was defined as an inflammatory condition affecting the surrounding tissues of a functioning osseointegrated implant and causing loss of supporting bone [16]. 10 males and 10 females, age range: 27–55. The inclusion criteria for the group selected by peri-implantitis were as follows: who had not periodontitis, presence of two implants with peri-implantitis and a minimum loading period of 12 months; peri-implant probing pocket depth (PIPD) > 5 mm; peri-implant presence of bleeding on probing (BOP) (with/without suppuration); radiographic signs of crestal bone loss (BL) in at least one area around an implant.

*Peri-mucositis group*: Peri-mucositis was defined as reversible inflammation of the soft tissues surrounding a functioning implant without loss of supporting bone [16]. 10 males and 10 females, age range: 28–57. The inclusion criteria for the group selected by peri-mucositis were as follows: who had not periodontitis, presence of at least two implants with peri-mucositis and a minimum loading period of 12 months; peri-implant probing pocket depth (PIPD) less than 5 mm; peri-implant presence of bleeding on probing (BOP) (with/without suppuration); had no radiographic signs of crestal bone loss (BL).

*Healthy controls*: 10 males and 10 females, age range: 26– 59. The control group consisted of subjects undergoing dental implant positioning who had not periodontitis or peri-mucositis and peri-implantitis; had a probing pocket depth (PIPD) equal to or less than 4 mm; and had no radiographic signs of peri-implant bone resorption.

### Saliva sampling

Before clinical measurements unstimulated whole saliva samples were obtained by expectorating into disposable tubes before clinical measurements early in the morning. Participants were asked to refrain from drinking anything other than water during the night and to not chew gum. Patients were instructed to collect saliva samples in the floor of their mouths by keeping their mouths open for five minutes, and then to spit out into five milliliter polypropylene tubes (ISOLAB SantrifugeTube, 078.02.001, Eschau, Germany). Comfortable and resting conditions were provided for the patients. After centrifuging saliva samples for 20 min at 1000xg to eliminate cell debris, the supernatants were then put into Eppendorf tubes and stored − 80 ºC until analysis [17].

### Measurement of Gpx activities

Glutathione peroxidase activity was measured according to Paglia and Valentine [18] 2.65 ml of 50 mM potassium phosphate buffer (pH 7.0) including 5 mM EDTA, 100 µl of GSH (150 mM), 20 µl of glutathione reductase (30 U/mL), 20 µl of NaN_3_ (0.12 M), 100 µl of NADPH (8 mM) and 50 µl of sample was mixed, and the tubes incubated for 30 min at 37 °C. The reaction was started by the addition of 100 µl of H2O2 solution (2 mM), mixed rapidly by inversion, and the conversion of NADPH to NADP was measured spectrophotometrically for 5 min at 340 nm.

### Measurement of SOD activities

Cu, Zn-superoxide dismutase activity was measured using the method described by Sun et al. [19]; 2.45 ml of assay reagent [0.3 mM xanthine, 0.6 mM Na_2_EDTA, 0.15 mM nitroblue tetrazolium (NBT), 0.4 M Na_2_CO_3_, 1 g/L bovine serum albumin] was combined with 0.5 mL of sample. Xanthine oxidase (50 µl, 167 U/L) was added to initiate the reaction and the reduction of NBT by superoxide anion radicals, which are produced by the xanthine-xanthine oxidase system, was determined by measuring the absorbance at 560 nm. Cu, Zn-superoxide dismutase activity was expressed as U/mL, where 1 U is defined as that amount of enzyme causing half-maximal inhibition of NBT reduction.

### Measurement of MDA levels

Malondialdehyde levels were measured in the clinical samples by the method of Jain et al. [20]. This method is based on the reaction of malondialdehyde with thiobarbituric acid to produce a complex that can be determined spectrophotometrically; 0.2 mL of sample were mixed thoroughly with 0.8 mL of phosphate buffered saline (pH 7.4) and 0.025 mL of butylated hydroxytoluene solution (0.88%).After addition of 0.5 mL of 30% ticholoroacetic acid, the samples were placed on ice for 2 h and then centrifuged at 2000 x g at 25 °C for 15 min. One mL of supernatant was mixed with 0.075 mL of 0.1 M EDTA and 0.25 mL of 1% tiobarbituric acid in 0.05 N NaOH. The samples were placed in boiling water for 15 min, cooled to room temperature, and the absorbance was determined at 532 nm.

### Measurement of 8-OHdG levels

The samples were centrifuged at 10,000 Å~g for 10 min, and levels of 8-OHdG in the supernatant were determined using a competitive enzyme-linked immunosorbent assay analysis (ELISA kit 8-OHdG Check, Highly Sensitive 8-OHdG Check, Japan Institute for the Control of Aging, Shizuoka, Japan ). Every measurement was completed in compliance with the guidelines provided by the manufacturer. There was no discernible cross-reactivity or interference in any of the duplicate samples. The percentages of variation for the inter-assay and intra-assay were 10%–%11 and 9%, respectively. The determination range was 0.125 to 200 ng/ml.

### Statistical analyses

IBM SPSS version 20 was used for statistical analysis. Descriptive statistics including measures of central tendency (mean, median) and dispersion (standard deviation, minimum, maximum), as well as frequencies (percentages and counts) were calculated. The normality of continuous variables was assessed using Q-Q plots, measures of skewness and kurtosis, and formal tests of normality (Kolmogorov-Smirnov and Shapiro-Wilk tests). The Kruskal Wallis test was chosen when the normal distribution criteria was not met and the ANOVA test was used when comparing continuous variables with more than two independent groups. Following an ANOVA analysis, the Tukey test for homogeneous variances and Tamhane’s T2 test for non-homogenous variances were performed as post-hoc tests. For post-hoc analyses, the Kruskal Wallis 1-way ANOVA (k samples) test was performed after the Kruskal Wallis test. The Pearson Chi-square test was utilized in 2 × 2 comparisons across categorical variables if the expected value was greater than 5, the chi-square Yates test was applied if the expected value was between 3 and 5, and the Fisher’s exact test was applied if the expected value was less than 3. If there was a normal distribution, the Pearson correlation was employed to compare two quantitative variables; if not, the Spearman correlation test was performed. Significant differences were defined as those with a p-value of less than 0.05.

## Results

### Demographic and Clinical Periodontal Parameters

A total of 60 patients, 30 men and 30 women were included. The mean ages were 40.30 ± 8.13, 42.76 ± 8.41, and 43.61 ± 7.66 for the healthy, peri-mucositis, and peri-implantitis groups, respectively. 318 implants were evaluated. 116 of these implants were healthy, 93 had peri-mucositis, and 109 had peri-implantitis. Significant differences were not observed in the genders and mean ages between groups. (*p* > 0.05) (Table 1).

**Table 1:** Demographic data

	Healthy Group *N*^a^: 20	Peri-mucositis *N*^b^: 20	Peri-implantitis *N*^c^: 20
**Gender (Male/Female)**	10 M/10F	10 M/10F	10 M/10F
**Age**	40,30 ± 8,13	42,76 ± 8,41	43,61 ± 7,66
**Number of implants**	116	93	109
**Maxilla**	49 (42%)	41 (44%)	45 (41%)
**Mandible**	67 (58%)	52 (56%)	64 (59%)

The healthy group had the lower PI and GI scores with a significant difference from the peri-mucositis group (*p* < 0.001) and the peri-implantitis group (*p* < 0.001). However, no difference was observed between the peri-mucositis and peri-implantitis groups (*p* > 0.05). The peri-implantitis group showed the highest PIPD value, which varied amongst the groups (*p* < 0.001). Although there was not a significant difference between the peri-mucositis and control groups (*p* > 0.05), the peri-implantitis group had a significantly higher BL score than the peri-mucositis and control groups (respectively *p* < 0.001; *p* < 0.001) (Table 2).

**Table 2:** Age and clinical parameters of the groups (mean ± SD)

	Healthy Group *N*^a^: 20	Peri-mucositis Group *N*^b^: 20	Peri-implantitis Group *N*^c^: 20	*P* value
**PI**	0.79 ± 0.24	2.04 ± 0.37	2.16 ± 0.43	***p < 0.001*** ^ab*^ ***p < 0.001*** ^ac*^ *p > 0.05* ^bc^
**GI**	0.67 ± 0.29	2.13 ± 0.43	2.27 ± 0.49	***p < 0.001*** ^ab*^ ***p < 0.001*** ^ac*^ *p > 0.05* ^bc^
**PD (mm)**	1.5 ± 0.2	1.7 ± 0.4	2.1 ± 0.3	*p > 0.05* ^ab^ *p > 0.05* ^ac^ *p > 0.05* ^bc^
**PIPD (mm)**	2.36 ± 0.30a	3.68 ± 0.56b	7.33 ± 0.79c	***p < 0.001*** ^ab*^ ***p < 0.001*** ^ac*^ ***p < 0.001*** ^bc*^
**BL (mm)**	0.15 ± 0.07a	0.22 ± 0.09 b	6.18 ± 0.84c	*p > 0.05* ^ab^ ***p < 0.001*** ^ac*^ ***p < 0.001*** ^bc*^

*ab comparison of mean values between healthy control group and peri-mucositis group;*

*ac comparison of mean values between healthy control group and peri-implantitis group;*

*bccomparison of mean values between peri-mucositis group; and peri-implantitis group*

*p < 0.001 Difference is significant

### Highest 8-OHdG levels in saliva was observed in peri-implantitis group

The levels of 8-OHdG in the saliva of the peri-implantitis, peri-mucositis, and control groups were 4.56 ± 0.65 ng/ml, 1.58 ± 0.40 ng/ml, and 1.32 ± 0.32 ng/ml, respectively. In comparison to the peri-mucositis and control groups, the peri-implantitis group had a significantly higher 8-OHdG level (*p* < 0.001; *p* < 0.001 respectively). Furthermore, there was no statistical difference between the control and peri-mucositis groups (*p* > 0.05) (Table 3).

**Table 3:** Laboratory findings between groups

	Healthy *N*^a^: 20	Peri-mucositis *N*^b^: 20	Peri-implantitis *N*^c^: 20	
8-OHdG (ng/ml)	1.32 ± 0.32	1.58 ± 0.40	4.56 ± 0.65	*p > 0.05* ^ab^ ***p < 0.001*** ^ac*^ ***p < 0.001*** ^bc*^
MDA(nmol/ml)	3.99 ± 0.56	4.14 ± 0.63	7.67 ± 0.88	*p > 0.05* ^ab^ ***p < 0.001*** ^ac*^ ***p < 0.001*** ^bc*^
SOD(U/ml)	2.81 ± 0.43	3.15 ± 0.58	5.16 ± 0.76	*p > 0.05* ^ab^ ***p < 0.001***^ac*^p < 0.001bc*
GPx(U/l)	82.16 ± 19.12	93.16 ± 20.65	55.16 ± 15.07	*p > 0.05* ^ab^ ***p < 0.001*** ^ac*^ ***p < 0.001*** ^bc*^

^ab^ *comparison of mean values between healthy control group and peri-mucositis group;*

^ac^ *comparison of mean values between healthy control group and peri-implantitis group;*

^bc^ *comparison of mean values between peri-mucositis group; and peri-implantitis group*

^*^p < 0.001 Difference is significant

### Highest MDA levels in saliva was observed in peri-implantitis group

The peri-implantitis, peri-mucositis, and control groups had saliva with MDA levels of 7.67 ± 0.88 nmol/ml, 4.14 ± 0.63 nmol/ml, and 3.99 ± 0.56 nmol/ml, respectively. The MDA level was significantly higher in the peri-implantitis group than in the peri-mucositis and control groups (*p* < 0.001; *p* < 0.001 respectively). Additionally, there was no statistically significant difference between the control group and the peri-mucositis group (*p* > 0.05) (Table 3).

### Highest SOD levels in saliva was observed in peri-implantitis group

SOD levels were 5.16 ± 0.76 U/ml, 3.15 ± 0.58 U/m, and 2.81 ± 0.43 U/m in the saliva of the peri-implantitis, peri-mucositis, and control groups, respectively. Compared to the peri-mucositis and control groups, the peri-implantitis group had a significantly greater SOD level (*p* < 0,001 and *p* < 0,001, respectively). Moreover, not a significant difference was seen between the groups with peri-mucositis and control (*p* > 0.05) (Table 3).

### Highest GPx levels in saliva was observed in peri-mucositis group

The saliva samples from the peri-implantitis, peri-mucositis, and control groups had GPx values of 55.16 ± 15.07 U/l, 93.16 ± 20.65 U/l, and 82.16 ± 19.12 U/l, respectively. The peri-implantitis group had considerably lower GPx levels than the peri-mucositis and control groups (*p* < 0,001 and *p* < 0,001, respectively), but there was no significant difference in GPx levels between the peri-mucositis and control groups (*p* > 0.05) (Table 3).

## Discussion

The human body has an antioxidant mechanism that keeps the balance between oxidation and reduction intact, and an imbalance that tilts in favor of ROS could result in more tissue damage [21]. In this study, we tested the hypothesis that there may be a disruption in the oxidant and anti-oxidant balance in peri-implant diseases. Our results show that 8-OHdG, MDA, and SOD values are significantly higher in the saliva of individuals with peri-implantitis, while GPx values are increased in individuals with peri-mucositis. This suggests that the oxidant and antioxidant balance is disrupted in peri-implant diseases.

Periodontitis and peri-implantitis share the same etiology and pathogenic mechanisms [1]. Some studies found that the inflammatory periodontium produced higher nitric oxide [22], superoxide anion levels, and myeloperoxidase activity [23]. Also, it has been shown that patients with chronic adult periodontitis have increased superoxide anion generation in their gingival fluid [24, 25]. When peri-implantitis is compared to periodontitis, there is a greater degree of tissue degradation and a higher concentration of neutrophilic granulocytes and macrophages [26, 27]. In a study, the peri-implantitis group showed greater infiltration of inflammatory cells and a decrease in collagen structure when compared to the periodontitis group [28]. It has been shown in animal models that both the gingiva and the peri-implant mucosa exhibit an increase in inflammatory cells and a loss of collagen during the early stages of inflammation; however, the lesions from peri-implantitis were larger and extended closer to the bone crest than those from periodontitis [29, 30].

Peri-implant diseases can also occur in implant patients without periodontitis and can lead to changes in the levels of biomarkers in saliva. In our study, we included periodontal healthy individuals in our study in order to eliminate oxidative stress caused by periodontitis, and we also took into account that all patients we included in our study had at least 4 implants. According to our results, 8-OHdG, MDA, SOD, and GPx values in saliva did not have a statistical difference between the health and peri-implant mucositis groups, while the peri-implantitis group was significantly different from these two groups. This showed that the oxidant and antioxidant levels in saliva changed with peri-implantitis.

8-OHdG level may be a marker in determining high oxidative stress [28]. An increased 8-OHdG level was observed with periodontitis in humans and mice. In our study, we detected higher 8-OHdG levels in the periimplantitis group compared to the other two groups. In another immunohistochemical study [31], an increased number of 8-OHdG-stained cells were observed in peri-implantitis and periodontitis tissue samples compared to healthy tissue samples. In our study, although the 8-OHdG level in saliva was measured using a different method, it supported this study.

The main lipid peroxidation by-product is MDA [32–34]. However, it has been found in patients with periodontal disease, where the crevicular fluid of periodontal teeth included higher levels of MDA [35, 36]. In our study, the MDA value was significantly higher in the peri-implantitis group than in the peri-mucositis and healthy groups. This showed that peri-implantitis has an etiopathology similar to periodontitis. On the other hand, in another study [15], in which MDA and MPO concentrations were evaluated in the saliva of patients with peri-implantitis in 4 to 5 implants, although the saliva concentrations of MDA and MPO were high in the peri-implantitis group, they did not show a significant difference with the healthy group. In another study, MDA level in peri-implant crevice fluid (PICF) was compared in peri-implantitis and healthy implants, and there was no difference between peri-implantitis and healthy PICFs [37]. The reason we found different results from this study may be due to our use of saliva samples instead of PICF and differences in the inflammatory responses of the participants. Another, in an in vitro peri-implantitis study [31], highest MDA level was observed in LPS-induced bone-marrow-derived mesenchymal stem cells (BMSCs) compared to the control group. This finding also supported our study.

Superoxide is an oxygen radical that is produced in inflammatory pathways and leads to the degradation of connective tissue. SOD is an antioxidant enzyme that fights this radical [36]. In a study comparing the SOD level in peri-implant space fluid (PICF) in peri-implantitis and healthy implants, no significant difference was found, although higher SOD levels were detected in patients with peri-implantitis [37]. The high level of SOD supported our study. With this, the reason no significant difference was found for MDA and SOD values in this study compared to ours may be due to the methods of collecting PICF.

The main functions of GPx are to neutralize hydrogen peroxide and stop the generation of free radicals that is dependent on H2O2 [38]. In our study, the lowest GPx level was observed in patients with peri-implantitis, while the highest level was in patients with peri-implant mucositis. There is not much literature on the effect of GPx in peri-implantitis. However, in an in vitro peri-implantitis study [31], lower GPx4 mRNA expression was observed in LPS-induced bone-marrow-derived mesenchymal stem cells (BMSCs) compared to the control group. Our findings and the results of this study are parallel to each other.

This study had some limitations. One of these was that 8-OHdG, MDA, SOD, and GPx activity were examined only in saliva samples, and PICF was not measured and compared. Another was that patients with only implants in the mouth were not selected, but patients with implants and teeth were also included in the study.

In conclusion, increased levels of oxidative stress in saliva may be associated with peri-implant disease. To precisely understand how oxidative damage occurs in peri-implant tissues, more research is necessary.

## Data Availability

No datasets were generated or analysed during the current study.
